# Interleukin 33, soluble suppression of tumorigenicity 2, interleukin 27, and galectin 3 as predictors for outcome in patients admitted to intensive care units

**DOI:** 10.1515/med-2023-0859

**Published:** 2023-12-21

**Authors:** Stevan Eric, Radica Zivkovic Zaric, Jasna Jevdjic, Svetlana Miletic Drakulic, Ivan Stanojevic, Danilo Vojvodic, Petar Arsenijevic, Bojan Stojanovic, Stefan Jakovljevic, Nenad Markovic, Milan Zaric, Petar Canovic, Jelena Nesic, Nenad Zornic

**Affiliations:** Department of Anesthesiology and Reanimation, University Clinical Center Kragujevac, Kragujevac, Serbia; Department of Surgery, Faculty of Medical Sciences, University Clinical Center Kragujevac, Kragujevac, Serbia; Faculty of Medicine of the Military Medical Academy, University of Defense, Belgrade, Serbia; Insitute for Medical Research, Military Medical Academy, Belgrade, Serbia; Department of Pharmacology and Toxicology, Faculty of Medical Sciences, University of Kragujevac, Kragujevac, Serbia; Department of Neurology, Faculty of Medical Sciences, University of Kragujevac, Kragujevac, Serbia; Department of Gynecology, Faculty of Medical Sciences, University of Kragujevac, Kragujevac, Serbia; Department of Biochemistry, Faculty of Medical Sciences, University of Kragujevac, Kragujevac, Serbia; Department of Endocrinology, University Clinical Center Kragujevac, Kragujevac, Serbia

**Keywords:** interleukin 33, galectin 3, interleukin 27, Intensive care units, outcome

## Abstract

Intensive care units (ICUs) are expert hospital areas that provide treatment and 24 h care for people who are very sick. Sepsis represents a serious, severe condition and it can lead to septic shock and multiple organ dysfunction syndromes and is one of the most common reasons for patients’ hospitalization in ICUs. We wanted to explore the prognostic values of interleukin (IL) 33, soluble suppression of tumorigenicity 2 (sST2), IL 27, and galectin 3 in critically-ill patients. We assumed that these parameters in combination or alone could predict mortality in ICU patients. This research represents a clinical non-randomized prospective study, performed at the Medical Military Academy, a tertiary care hospital in Belgrade, Serbia. The patients were divided in four groups: patients with sepsis (peritonitis, pancreatitis, trauma) and patients without sepsis (trauma). Total number of patients enrolled in the study was 151 and average years of patients were 56.48. The values greater than the cut-off were the predictors of mortality. The IL-33, IL-27 as well as galectin-3 can successfully predict the outcome of critically-ill patients in ICUs. The sST2, cannot predict death in critically-ill patients as a single prognostic factor. However, the combination of at least two biomarkers: IL-33, sST2, IL-27, and galectin-3, gives very significant results in predicting the outcome in patients admitted to ICUs.

## 1 Introduction

Intensive care units (ICUs) are expert hospital areas that provide treatment and 24 h care for people who are very sick. Sepsis represents a serious, severe condition and it can lead to septic shock and multiple organ dysfunction syndromes and is one of the most common reasons for patients’ hospitalization in ICUs [1,2].

Sepsis is a significant public health problem with high morbidity and mortality. In patients with sepsis, the morbidity rate amounts to 29.5% in the hospital and 47% in the ICUs. Furthermore, the death rate in patients with sepsis is about 25.8% in the ICUs and 35.3% in the hospital [1]. Microbiological culture represents a gold standard for diagnosing sepsis, but considering that it takes time for the findings to arrive, which could be fatal for the patient, the need for other biomarkers for the diagnosis and prognosis of sepsis arose [3].

For now, procalcitonin is the most common diagnostic biomarker for sepsis, as well as presepsin [4,5]. Other biomarkers are also mentioned like interleukin (IL) 33. IL-33 is a pro-inflammatory cytokine of the IL-1 family.

Extracellular IL-33 is a ligand for the suppression of tumorigenicity 2 (ST2) receptor and has a vital role at the beginning of severe local inflammation as well as in the guideline of adaptive immune responses. The IL-33/ST2 system has also a significant role in the pathogenesis of acute and chronic inflammatory, autoimmune, and allergic illnesses, i.e., sepsis, asthma, anaphylaxis, rheumatoid arthritis, chronic obstructive pulmonary disease, etc. [6]. ST2 is an associate of the IL-1 receptor family. Soluble ST2 (sST2) is a developing, new biomarker associated with cardiac fibrosis, and its utility as a predictive indicator for heart failure has established rising attention [7]. Binding of IL-33 to sST2 inhibits the useful and defensive properties of IL-33 on the heart. The beginning of the IL-33/transmembrane isoform of the ST2 signaling pathway is also known in other conditions such as inflammation. Exogenous hazard signals such as bacteria and endotoxins motivate inflammatory cytokine excretion, improve sST2 creation and therefore weaken immune responses in the organism [7].

IL 27 is formed by antigen-presenting cells open to inflammatory inducements and other settings. IL-27 encourages the production of naive CD4+ T cells and causes both pro-inflammatory and anti-inflammatory responses and according to some authors, it could be the representative biomarker for sepsis [1,8].

A family of β-galactoside-binding lectins with ≥1 evolutionary preserved carbohydrate-recognition domain is known as galectins. Galectin-3 is extensively expressed in human tissues, as well as in all immune cells (macrophages, monocytes, dendritic cells, eosinophils, mast cells, natural killer cells, and activated T and B cells), epithelial and endothelial cells as well as in sensory neurons [9,10]. Jevdjic et al. suggested galectin-3 as a novel biomarker for sepsis [11].

According to serious cellular and metabolic abnormalities in sepsis, numerous biomarkers if acted together may represent an objective guide for the prognosis of patients with severe sepsis as well as in patients with trauma. According to the literature, there is no information about the prognostic values of IL-33, sST2, IL-27, and galectin 3 in critically-ill patients. Therefore, we wanted to explore the prognostic values of IL-33, sST2, IL-27, and galectin-3 in these patients. We assumed that these parameters in combination or alone could predict mortality in ICU patients.

## 2 Methods

This research represents a clinical non-randomized prospective study, performed at the Medical Military Academy, tertiary care hospital in Belgrade, Serbia in the period from January 2021 until March 2022. The study was approved by the Ethical Committee of Medical Military Academy. Informed consent was obtained from the patient or first-degree relative. Patients hospitalized in ICUs were included in the study.

Inclusion criteria were (1) adults patients, both male and female, (2) patients with a diagnosis of acute pancreatitis, peritonitis, or trauma, (3) patients with sepsis (two criteria of the systemic inflammatory response plus the above-mentioned diagnosis), (4) patients with injury severity score (ISS) above 10, and (5) critical patients with Acute Physiology and Chronic Health Evaluation score (APACHE II) above 15. Exclusion criteria were (1) secondary sepsis and/or septic shock with an underlying illness other than severe peritonitis, pancreatitis, or trauma, (2) pre-existing immunodeficiency, (3) long-term SICU stay before norms fulfilment, and (4) malignant disease of any source. Patients were divided in four groups according to the reason for admission: patients with sepsis (peritonitis, pancreatitis, trauma) and patients without sepsis (trauma).

The Sequential Organ Failure Assessment (SOFA) score, the Simplified Acute Physiology Score (SAPS) II, and the APACHE II score were considered and verified within the first 24 h after admittance to the ICU as well as blood sample of each patient for further examination. We examined the levels of IL-33, sST2, IL-27, and galectin-3. We also examined each blood sample for hemoculture.

A blood sample was obtained from each patient within 24 h after admission from the arterial or central venous line. Approximately 8 mL of blood was drained into a serum centrifuge tube and centrifuged at 4°C at 2,500 rpm for 15 min in order to separate blood elements and serum. Then, a sample of blood serum was collected for each patient and stored at −70°C for future examination.

Levels of IL-33 were measured using the Human IL-33 DuoSet^®^ ELISA (R & D Systems, Minneapolis, MN, USA) according to the producer’s instructions (sensitivity 1.65 pg/mL) [6]. Levels of sST2 were measured by the Presage ST2 Assay (Critical Diagnostic, San Diego, CA, USA). The mouse monoclonal anti-human sST2 antibodies in combination with enzyme-linked immunosorbent assay was used to evaluate sST2 levels [12]. IL-27 was measured by an enzyme immune assay (Human IL-27 ELISA, Kit: Duo Set [R&D]) according to the manufacturer’s instructions [13]. The concentration of galectin-3 was determined using the Quantikine Human Galectin-3 Immunoassay ELISA test (R&D Systems Europe Ltd, UK) [11].

The basic subdivision of patients was done according to the outcome (survival and non-survival). One of the important hypotheses was based on the correlation between outcome and serum galectin-3 (soluble β-galactosidase-binding lectin) level. A preliminary analysis was done and the expected minimal difference in galectin-3 mean values between survivors and non-survivors was estimated to be 80%. [11]. The calculated number of patients was at least 128 (total sample size) according to the test power of 0.8 (80%) and the alpha probability of 0.05. Due to high values of standard deviations (SDs) and two-independent groups, the Wilcoxon test model was used (statistical software package GPower 3.1).

Complete statistical analysis of data was done with the statistical software package, SPSS Statistics 18. Most of the variables were presented as the frequency of certain categories, while the statistical significance of differences was tested with the Chi-square test.

In the case of continuous data, variables were presented as mean value ± SD or median if the distribution of the data was not normal. The Kolmogorov–Smirnov test was used for the evaluation of the distribution of data. Statistical significance between the groups was tested by Kruskal–Wallis or Mann–Whitney test. Spearman correlation analyses were used to establish the relation between parameters.

Receiver operating characteristic (ROC) curves were constructed to determine the sensitivity and specificity of mediators for the prediction of outcome, diagnosis, and infection. The Youden’s index, is the difference between the true positive rate and the false positive rate, was used.

Maximizing this Youden’s index allowed us to determine an optimal cut-off point from the ROC curve independently. All the analyses were estimated at *p* < 0.05 level of statistical significance.

## 3 Results

Total number of patients enrolled in the study was 151 and average years of patients were 56.48. All demographic characteristics of the patients as well as median serum concentration of IL-33, sST2, IL-27, and galectin-3 are shown in Table 1. According to the reason for the hospitalization there were 131 patients with sepsis (76 patients with peritonitis, 22 patients with pancreatitis, and 23 trauma patients) and 30 patients without sepsis. Hospital mortality was 49.7% and it was higher in female patients (66.7%).

**Table 1:** Characteristics of the patients included in the study

Variable	All patients (*N* = 151)
Age (average, range)	56.48 (17–91)
**Sex,** ***n*** **(%)**	
Male	97 (64.2%)
Female	54 (35.8%)
SAPS II score, mean ± SD	52.45 ± 7.24
APACHE II score, mean ± SD	22.69 ± 3.98
SOFA score, mean ± SD	7.45 ± 2.54
Length of ICU stay, mean ± SD, range (days)	14 ± 10 (2–168)
Length of hospital stay, mean ± SD, range (days)	62 ± 28 (2–338)
**Reason for ICU admission,** ***n*** **(%)**	
Sepsis due to	
Peritonitis	76 (50.3%)
Pancreatitis	22 (14.6%)
Severe trauma	23 (15.2%)
Severe trauma (ISS 26.43 ± 8.56)	30 (19.9%)

**Figure 1:**
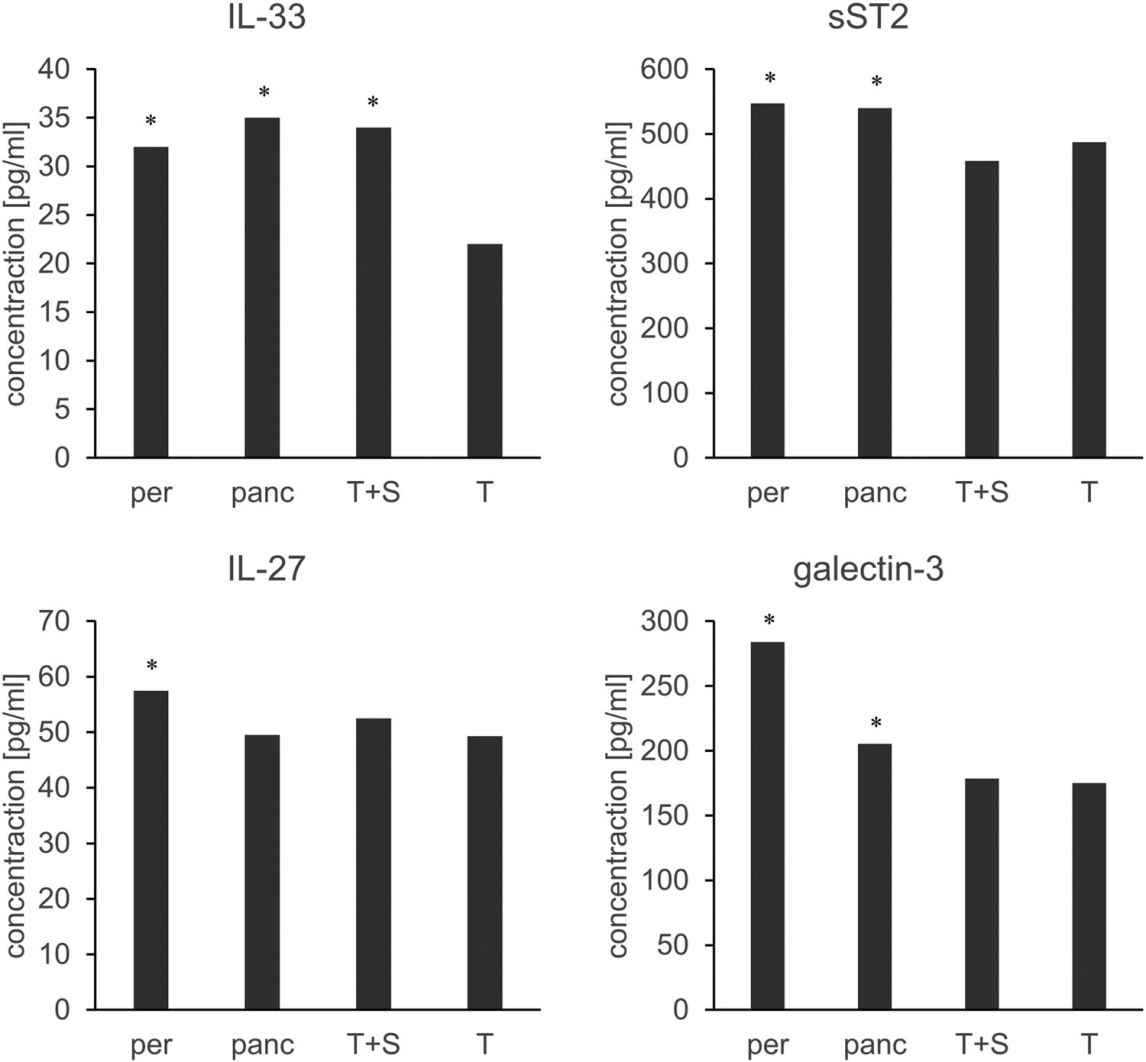
Average values of IL-33, sST2, IL-27, and galectin-3 in the serum of the patients admitted to ICU with sepsis caused by peritonitis (per), pancreatitis (panc), trauma (T + S), or with trauma without sepsis (T). The results are presented as medians, *p < 0.05 in comparison to the group of patients with trauma.

The results of our research showed that the values of IL-33 were significantly higher in the group of patients with sepsis caused by peritonitis, pancreatitis, and trauma compared to the control population of patients who had only trauma (*p* < 0.05, Figure 1). The values of sST2 were significantly higher in the group of patients with sepsis caused by peritonitis and pancreatitis compared to the control population of patients who had only trauma, while the values of IL-27 were significantly increased in the group of patients with sepsis caused by peritonitis compared to the control population of patients who had only trauma (*p* < 0.05, Figure 1). The values of galectin-3 were significantly increased in the group of patients with sepsis caused by peritonitis and pancreatitis compared to the control population of patients who had only trauma (*p* < 0.05, Figure 1).

**Figure 2:**
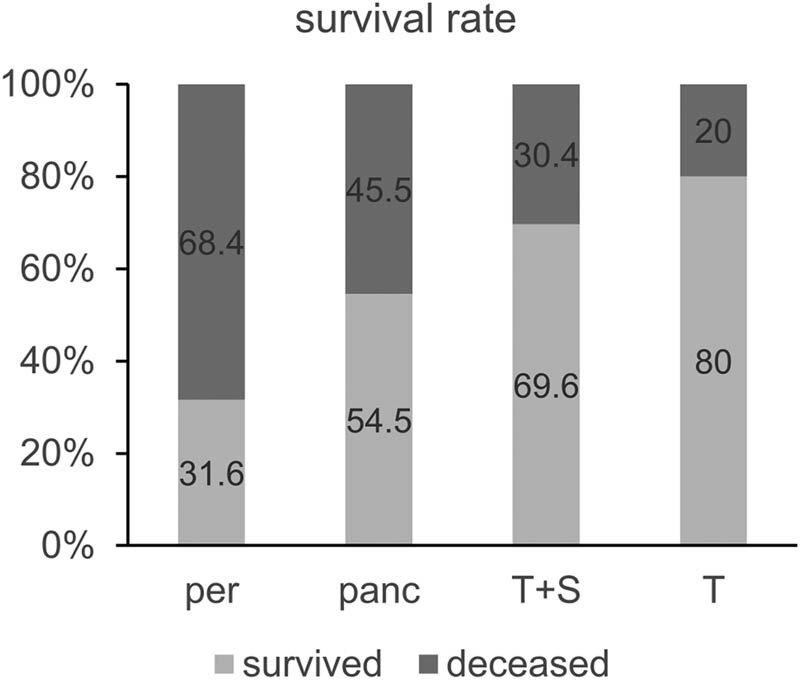
Survival rate of the patients admitted to ICU with sepsis caused by peritonitis (per), pancreatitis (panc), trauma (T + S), or with trauma without sepsis (T). The results are presented as relative values.

Patients who had sepsis caused by peritonitis, pancreatitis, or trauma had a significantly decreased survival rate compared to the control group of patients who had only trauma (*p* < 0.05, Figure 2). Patients who had peritonitis diagnosed, had a 3.4-fold increased occurrence of death, compared to trauma patients. Similarly, patients who had pancreatitis diagnosed had a 2.2-fold increased occurrence of death, compared to trauma patients.

**Figure 3:**
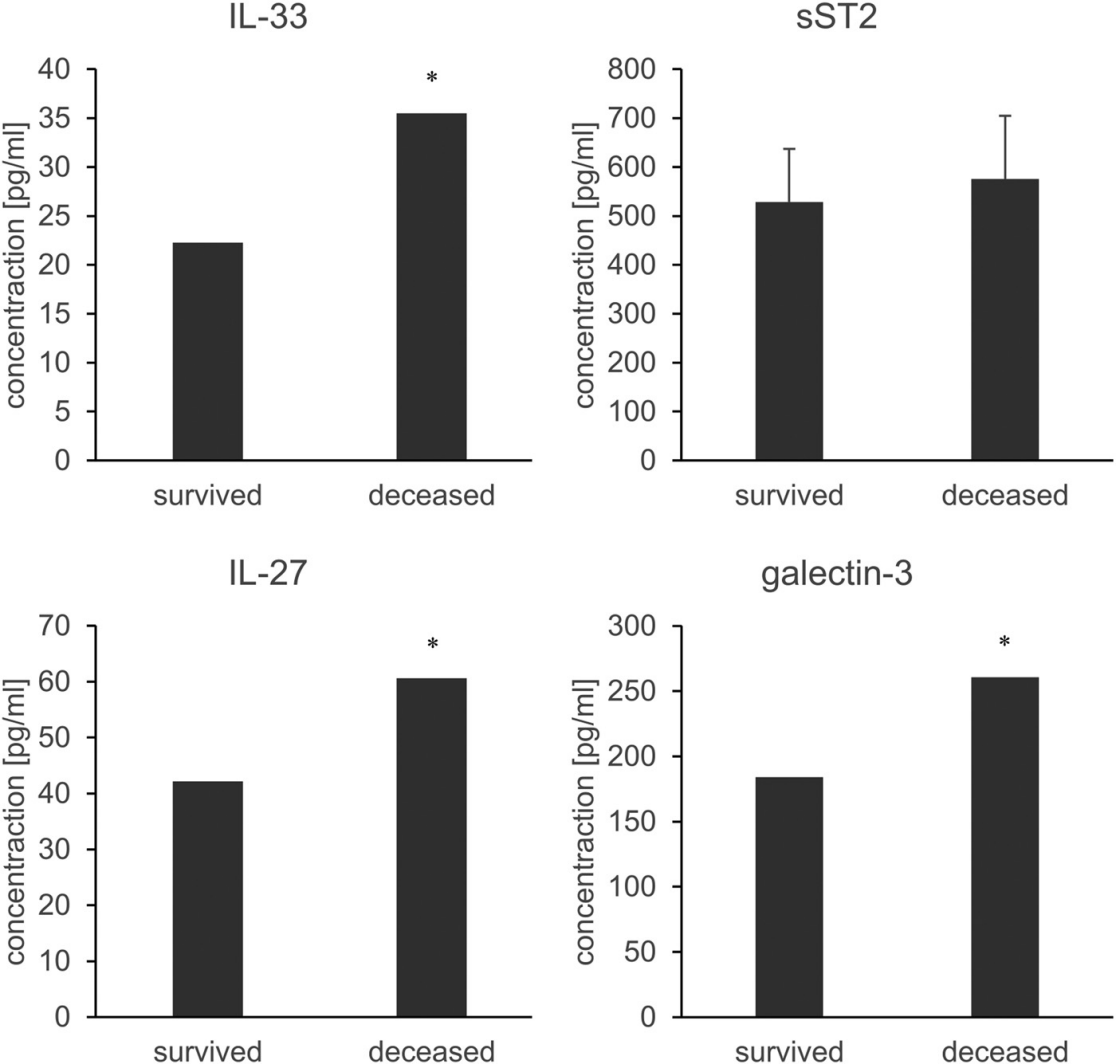
Average values of IL-33, sST2, IL-27, and galectin-3 in the serum of the survived and deceased patients. The results are presented as medians (for IL-33, IL-27, and galectin-3) or mean ± SD (sST2). *p < 0.05 between groups.

After that, we determined the average values of the examined parameters (IL-33, sST2, IL-27, and galectin-3) in the group of patients who had a satisfactory outcome, i.e., who survived, and we compared the values of these parameters to the values of the same parameters in the group of deceased patients. The results showed that the values of IL-33, IL-27, and galectin-3 were significantly increased in the group of deceased patients compared to the values of IL-33, IL-27, and galectin-3 of surviving patients (*p* < 0.05, Figure 3). There was no statistically significant difference between the values of sST2 in surviving patients compared to the values of sST2 in deceased patients (*p* > 0.05, Figure 3). All biomarkers but sST2 were good predictors of outcome.

Afterward, we calculated cut-off values for each examined biomarker (IL-33, sST2, IL-27, and galectin-3). Values greater than the cut-off were the predictors of mortality. The cut-off values for IL-33, sST2, IL-27, and galectin-3 were 30.5, 599.0, 42.47, and 258.93, respectively. The cut-off values for each biomarker, Youden index, sensitivity, and specificity are presented in Table 2.

**Table 2:** Cut-off values for IL-33, sST2, IL-27, and galectin-3 as predictors of mortality

	Cut-off (ng/mL)	Youden index	Sensitivity (%)	Specificity (%)
IL-33	30.5	0.26	63.8	62.2
sST2	599.0	0.158	29.3	86.5
IL-27	42.47	0.168	77.6	39.2
Galectin-3	258.93	0.203	50	70.3

**Figure 4:**
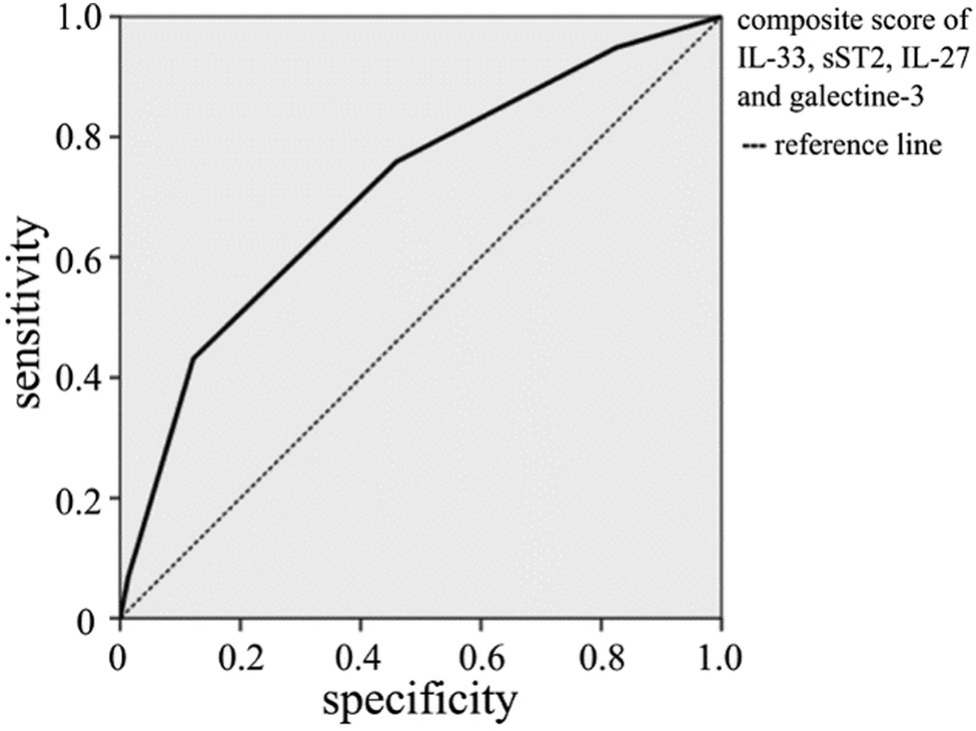
ROC curve of the prediction of mortality for composite scores of IL-33, sST2, IL-27, and galectine-3.

The biomarker with the best sensitivity and specificity as predictor of mortality was IL-33. The next step of our research was to estimate if values above the cut-off for two or more parameters could improve the prediction for outcomes in patients admitted to ICUs. So, in order to increase the overall sensitivity and specificity of these biomarkers combined, we determined their composite score. For a composite score of 1.5, the calculated sensitivity was 76%, specificity was 54%, and Youden index was 0.3. The results showed that two of the tested cytokines (IL-33, sST2, IL-27, and galectin-3) must have values above the cut-off to achieve a statistically highly significant predictive estimate of the outcome for the patients admited in the ICUs (Figure 4).

## 4 Discussion

Our study included 151 ICU patients with male predomination (97 vs 54). The average length of treatment in the ICU was 14 ± 10 days with an average length of hospital stay of 62 ± 28 days. Most of the patients had sepsis due to peritonitis (*n* = 76). Number of Patients hospitalized because of trauma were 30. Of From the tested biomarkers, IL-33, IL-27, and galectin-3 were a good predictors of outcome. A combination of at least two biomarkers gave the best results in predicting the outcome with the calculated sensitivity 76%, specificity 54%, and Youden index 0.3.

The domination of male patients is following the results of other studies [14,15]. It was concluded that there was a higher morbidity and mortality rate from sepsis in men, and this seems to be associated with differences in respiratory tract infection frequency and IL-6 plasma levels, among the genders [16]. SAPS II score in our study was at an average of 52.45. The SAPS II helps to calculate in-hospital mortality, thanks to the gathering of 17 variables at ICU admission, without considering the aim for admission [17]. APACHE II score has a mean value of 22.69 which is following other research, for the patients admitted in the ICU [18]. SOFA score in our study had a mean value of 7.45. The SOFA value over 72 h has an important confident connection to in-hospital mortality [19].

According to our results IL-33 represented significant predictors for the outcome in critically-ill patients. sST2 had limited prognostic value for the overall outcome as a single prognostic factor. Moreover, IL-27 as well as galectin-3 were also significant predictors for the outcome in critically-ill patients.

IL 33 showed solid sensitivity (63.8) and specificity (62.2). Our results were opposite to the results of other studies because they suggested that IL-33 values were decreased in patients who died from sepsis in comparison to patients who survived [20,21]. Also, according to our results, sST2 was not a significant predictor of outcome in ICU patients. sST2 behaves as a trap for IL-33, so since in our study, it did not prove to be significant, we can explain why increased IL-33 can be a predictor of mortality according to our results. In our population, 30 patients with trauma were included, and IL-33 levels tend to increase following trauma. Therefore, this may be another reason for such difference in the results of our study in comparison to previously published results [22].

For IL-27 values greater than 42.47 ng/mL on the first day of hospitalization, can predict mortality in ICUs patients. The sensitivity of this claim was 77.6 and the specificity was 39.2.

Wang et al. showed that in the meta-analysis, the sensitivity was about 0.84 and the specificity was about 0.71, demonstrating a satisfactory overall diagnostic accuracy of IL 27 [1].

Galectin-3 value greater than 258.93 ng/mL on the first day of hospitalization, was a good predictor of mortality which is in accordance with the results of other studies [5,11,23]. The sensitivity of this analysis was 50 and the specificity was 70.3. It was determined that galectin-3 acts as alarmin and exhibits immune-triggering properties such as motivation of oxidative burst in neutrophils and inflammatory cytokine construction in macrophages. According to Mishra et al., galectin-3 plays a pathogenic character as an alarmin to worsen the inflammatory reaction through pulmonary infection with some bacteria and contributes to the progress of sepsis [24].

Combination of at least two biomarkers gave the best results in predicting the outcome of ICU patients. Values higher than the cut-off, including sST2, can predict death in critically-ill patients.

The limitation of our study was the relatively small number of patients; therefore, it remains a task for future studies to further examine our claims on a larger number of patients.

The conclusion of our study was that IL-33, IL-27, as well as galectin-3 can successfully predict the outcome of critically-ill patients in ICUs. sST2, cannot predict death in critically-ill patients as a single prognostic factor. However, the combination of at least two biomarkers: IL-33, sST2, IL-27, and galectin-3, gives very significant results in predicting the outcome in patients admitted to ICUs.
